# Multimorbidity in the elderly in China based on the China Health and Retirement Longitudinal Study

**DOI:** 10.1371/journal.pone.0255908

**Published:** 2021-08-05

**Authors:** Xiaorong Guo, Benhua Zhao, Tianmu Chen, Bin Hao, Tao Yang, Huimin Xu

**Affiliations:** 1 Department of Vascular Surgery, Shanxi Bethune Hospital, Shanxi Academy of Medical Sciences, Tongji Shanxi Hospital, Third Hospital of Shanxi Medical University, Taiyuan, Shanxi, China; 2 State Key Laboratory of Molecular Vaccinology and Molecular Diagnosis, School of Public Health, Xiamen University, Xiamen, Fujian, China; Universidade Federal de Pelotas, BRAZIL

## Abstract

This study aimed to investigate the spatial distribution and patterns of multimorbidity among the elderly in China. Data on the occurrence of 14 chronic diseases were collected for 9710 elderly participants in the 2015 waves of the China Health and Retirement Longitudinal Study (CHARLS). Web graph, Apriori algorithm, age-adjusted Charlson comorbidity index (AAC), and Spatial autocorrelation were used to perform the multimorbidity analysis. The multimorbidity prevalence rate was estimated as 49.64% in the elderly in China. Three major multimorbidity patterns were identified: [Asthma/Chronic lungs diseases]: (Support (S) = 6.17%, Confidence (C) = 63.77%, Lift (L) = 5.15); [Asthma, Arthritis, or rheumatism/ Chronic lungs diseases]: (S = 3.12%, C = 64.03%, L = 5.17); [Dyslipidemia, Hypertension, Arthritis or rheumatism/Heart attack]: (S = 3.96%, C = 51.56, L = 2.69). Results of the AAC analysis showed that the more chronic diseases an elderly has, the lower is the 10-year survival rate (*P* < 0.001). Global spatial autocorrelation showed a positive spatial correlation distribution for the prevalence of the third multimorbidity pattern in China (*P* = 0.032). The status of chronic diseases and multimorbidity among the elderly with a spatial correlation is a significant health issue in China.

## Introduction

Despite many unpredictable hygiene challenges can be life threatening and cause the population decline, such as climate change caused by the extreme weather, emerging infectious diseases such as SARS, COVID-19, and superbugs, the population is aging at an accelerating rate and most people will live beyond the age of 60, with far-reaching impact on health systems, health workers, and health budget in China and around the world.

Aging is often accompanied by multimorbidity or contemporaneous occurrence of a variety of chronic diseases [1]. The World Health Organization (WHO) defined multimorbidity as the coexistence of two or more chronic conditions in the same individual. Various studies have reported that elderly patients with multimorbidity are at higher risk of death, longer hospital stay, poorer quality of life, and poorer physical function than those with a single chronic disease [2–5]. The burden caused by multimorbidity is multifaceted, and impacts medical and health resources, families, and caregivers [6]. Barnett K et al. [7] reported that patients with multimorbidity had lower functional status, lower quality of life, and poor health outcomes, along with higher rates of outpatient visits and hospitalizations than patients with a single chronic disease.

International study of multimorbidity, for its definition generally agree with the WHO, just a few research defined as people with three or more chronic diseases at the same time, the study population are usually derived from the general population or primary care clinical practice, with a large sample size and strong representativeness [8–10]. However, in China, due to its vast territory and large population, and the different medical systems used in different regions, it is difficult to achieve large-scale cross-sectional survey, and the types and quantities of diseases included in the study are also different. The existing research in China also has very limited information on the elderly population. Patterns of multimorbidity refer to the classification of chronic conditions into different combinations based on associations between the chronic conditions [11]. Different multimorbidity patterns can identify the common risk factors, pathogenesis, and/or drug interaction among the chronic diseases [12–14]. A systematic review published in 2015 showed that the prevalence of multimorbidity in the elderly in China reported in the literature varies widely between 6.4% and 76.5% [15]. This is probably due to differences in the methodology used for each report, including sources of data, sample size, accuracy of data collection, and analytical approaches, especially the number and categories of chronic diseases. The prevalence of multimorbidities was 13.80% in a cross-sectional survey of 17867 adults aged 45 or older in Shandong, China [16]. A cross-sectional survey of middle-aged and elderly people in a Chinese community showed that the prevalence of multimorbidities was 20.8%, and the association between hypertension and diabetes mellitus was the strongest [17].

The pathogenesis of chronic disease is complicated, and researchers have gradually realized that the occurrence of chronic diseases is not only related to heredity and unhealthy lifestyle, but also to the human environment in terms of geographical location, environment, and climate, as well as the levels of pollution caused by various harmful substances [18, 19]. Therefore, it is necessary to analyse the distribution of multimorbidity patterns by its association with the geographical factors of the environment.

In this study, the 2015 waves of China Health and Retirement Longitudinal Study (CHARLS) database were adopted, and methods such as Apriori algorithm and spatial autocorrelation were used to explore the prevalence and patterns of multimorbidity among the elderly in China, as well as the spatial distribution of multimorbidity. Additionally, we aimed to further promote the prevention and control of chronic diseases and provide data for the implementation of effective management of multimorbidity in the future.

## Materials and methods

### Chronic disease in the Chinese elderly data source

Relevant data for 9786 participants aged 60 year and above were selected from the 2015 waves of CHARLS (See S1 File for details of the CHARLS database). Presence of chronic disease conditions was determined by reviewing the Health Status and Functioning part in response to the following question: “Have you been diagnosed with [conditions listed below, read one by one] by a doctor?” 1. Hypertension; 2. Dyslipidemia (elevation of low-density lipoprotein, triglycerides (TGs), and total cholesterol, or a low high density lipoprotein level); 3. Diabetes or high blood sugar; 4. Cancer or malignant tumour (excluding minor skin cancers); 5. Chronic lung diseases, such as chronic bronchitis, emphysema; 6. Liver disease (except fatty liver); 7. Heart attack, coronary heart disease, angina, congestive heart failure, or other heart problems; 8. Stroke; 9. Kidney disease; 10. Stomach or other digestive disease; 11. Emotional, nervous, or psychiatric problems; 12. Memory-related disease; 13. Arthritis or rheumatism; 14. Asthma. By comparing the survey data in 2011 and 2013, incomplete and abnormal data were deleted. Finally, the basic information and chronic disease information of 9,710 elderly aged 60 years and above were selected and the database was established.

### Ethics

Raw data were obtained free of charge from the website of the CHARLS (http://charls.pku.edu.cn/zh-CN). The data are publicly available. They can be downloaded with personal information removed.

### Geographic data source

Based on the China province (municipality) electronic map of 1:1,000,000 (http://bzdt.ch.mnr.gov.cn/browse.html?picId=%224o28b0625501ad13015501ad2bfc0240%22), ArcGIS 10.2 (Environmental Systems Research Institute, Redlands, California, USA) software was used to correlate the prevalence of chronic diseases and multimorbidity of the elderly in China with the electronic map, and to establish the database.

### Geospatial and statistical methods

Descriptive statistics were used to describe the characteristics of the chronic diseases and multimorbidity in the elderly population of China. Chi-square test was used to compare prevalence of chronic diseases and multimorbidity between urban and rural and between sexes. Next, the web graph was used to estimate the potential association between chronic diseases based on an Apriori algorithm that was adopted for this purpose. Age-adjusted Charlson comorbidity (AAC) index was used to assess the impact of multimorbidity in elderly. Finally, we used spatial autocorrelation to explore the spatial distribution characteristics of multimorbidity.

### Web graph

In the web graph, each chronic disease is shown as a dot. If the patient has two chronic diseases at the same time, the dots of the two chronic diseases are connected by line segments. The thickness of the line segment is determined by the frequency of the two chronic diseases being affected at the same time. The thicker the line is, the stronger the link strength will be. Thus, the correlation strength can be roughly reflected by different types of lines. Dotted lines represent weak correlation; normal thin lines and thick lines represent moderate and strong correlations, respectively.

### Association rule

There are many effective algorithms of association rules, among which the most classical one is Apriori algorithm. Indexes of evaluation include Support, Confidence, and Lift. Support (S): A→B represents the percentage of A∩B that is contained in transaction database D, that is, the probability of *P(A∩B)*. Confidence (C): is the percentage of B contained in D when A is also contained in D, that is, the probability of *P(B│A)*. Lift (A): is the ratio of the conditional probability of the occurrence of B to the unconditional probability of occurrence of B in the case of A, that is, P(B│A)│P(B)*P(A). The Lift is divided by 1. When the Lift >1, it indicates that there is a positive correlation between A and B and that there is multiple possibility of the occurrence of B under the condition of A. When Lift = 1, it means that the association rule reflects a common phenomenon. These three indices are indispensable, and single use of any one of them will result in wrong conclusions.

Based on the characteristics of the current survey of chronic diseases, the existence of antecedent and association rules cannot prove the causal relationship; thus, it is called Left-hand-side and Right-hand-side to avoid confusion. In this study, the screening conditions of association rules were set as follows: the min-support was 3.0%, the min-confidence was 50.0%, and the left-hand-side was 1. In combination with the research sample, this study further analysed the left-hand-side for 2 and 3 (according to the institute set minimum support, confidence, when left-hand-side >3, the number of association rules is equal to the left-hand-side = 3), to explore the correlation between a variety of chronic diseases.

### Age-adjusted Charlson comorbidity index

AAC included 19 diseases, which were weighted 1, 2, 3, and 6 points according to their severity. The age-adjusted score is 1 point for the age of 50 to 59 years, and 1 point is added for every 10 years. Finally, the 10-year survival rate was calculated according to the corresponding formula.

### Spatial autocorrelation

Spatial autocorrelation mainly includes global spatial autocorrelation and local spatial autocorrelation.

Global spatial autocorrelation analysis mainly uses Moran’s I coefficient to reflect the degree of spatial aggregation of attribute variables in the whole area. Moran’s I >0 indicates that the attribute variables have positive correlation in the study area; Moran’s I <0 indicates that the attribute variables have negative correlation in the study area, and Moran’s I = 0 suggests that the attribute variables are randomly distributed in the study area and there is no autocorrelation.

Local spatial autocorrelation is used to analyse the spatial relationship between adjacent regions, and Moran’s I along with LISA (local indicators of spatial autocorrelation) is used to reflect the spatial aggregation degree of attribute variables in local regions. For the case of area unit I, Ii >0 indicates high-high type (unit attribute value is high, the adjacent unit attribute value is high) or low-low type (unit attribute value is low, the adjacent unit attribute value is low), Ii <0 indicates high-low type (unit attribute value is high, the adjacent unit attribute value is low) or low-high type (unit attribute value is low, adjacent unit attribute value is high).

### Statistic software

Stata 13.0 was used for sorting and screening data. Descriptive statistics analyses and chi-square test were performed by using SPSS 19.0(IBM SPSS, IBM Corp, Armonk, NY, USA). IBM. SPSS Clementine 12.0 was used to prepare the web graph, and Apriori algorithm. Spatial autocorrelation analysis was performed in OpenGeoDa9.5.

## Results

### Prevalence of chronic diseases and multimorbidity

As shown in Table 1, 39.6% of the study population lived in urban area, and 49.4% were male. The highest prevalence rate for chronic diseases was calculated as 38.6% for arthritis or rheumatism, followed by hypertension (33.31%), while the prevalence rate of multimorbidity in this elderly population was 49.46%.

**Table 1:** The prevalence of chronic disease and multimorbidity.

Chronic diseases	Urban (%)			Rural (%)			Total (%)			Urban-rural P	Gender P
	Male	Female	Total	Male	Female	Total	Male	Female	Total		
Hypertension	34.17	38.36	36.32	28.66	33.99	31.33	30.81	35.75	33.31	<0.001	<0.001
Dyslipidemia	20.00	21.20	20.62	10.27	13.57	11.92	14.06	16.64	15.36	<0.001	<0.001
Diabetes or high blood sugar	13.64	15.33	14.51	6.76	8.56	7.66	9.44	11.28	10.37	<0.001	0.003
Cancer or malignant tumor	1.82	1.72	1.77	0.92	1.26	1.09	1.27	1.45	1.36	0.005	0.455
Chronic lung diseases	12.99	9.41	11.15	15.25	11.15	13.2	14.37	10.45	12.39	0.003	<0.001
Liver disease	3.90	2.88	3.38	3.75	2.97	3.37	3.81	2.93	3.37	0.016	0.956
Heart attack	20.27	26.57	23.50	13.61	19.09	16.35	16.20	22.10	19.19	<0.001	<0.001
Stroke	5.99	2.94	4.42	3.96	3.07	3.51	4.75	3.01	3.87	0.023	<0.001
Kidney disease	9.63	6.83	8.19	8.70	6.27	7.49	9.06	6.50	7.73	0.205	<0.001
Stomach or other digestive disease	18.50	25.66	22.18	23.00	29.66	26.33	21.25	28.05	24.69	<0.001	<0.001
Emotional, nervous, or psychiatric problems	1.07	1.32	1.20	1.09	1.94	1.52	1.08	1.69	1.39	0.185	0.011
Memory-related disease	4.22	3.90	4.06	2.93	3.14	3.04	3.44	3.44	3.34	0.007	0.987
Arthritis or rheumatism	26.42	41.35	34.09	36.17	47.08	41.63	32.37	44.77	38.64	<0.001	<0.001
Asthma	6.84	3.90	5.33	7.81	5.63	6.72	7.44	4.93	6.17	0.005	<0.001
Multimorbidity	48.45	55.87	52.26	43.77	51.48	47.63	45.59	53.25	49.46	<0.001	<0.001

The Chi-square test was used to compare the prevalence of chronic diseases and multimorbidity between urban and rural and between genders. Except for cancer and other malignant tumors and memory-related diseases, the differences of prevalence of chronic diseases and multimorbidity was statistically significant between genders, and the prevalence of female was generally higher than that of male. The prevalence of chronic lung diseases, stroke, kidney disease, and asthma of male was higher than that of female. In addition to liver disease, kidney disease, emotional, nervous, or psychiatric problems, there were significant differences in chronic diseases and multimorbidity between urban and rural, and the prevalence urban is generally higher than that in rural, and the prevalence of stomach or other digestive disease, emotional, nervous, or psychiatric problems, arthritis or rheumatism and asthma is higher in rural than in urban.

### Web graph

**Fig 1:**
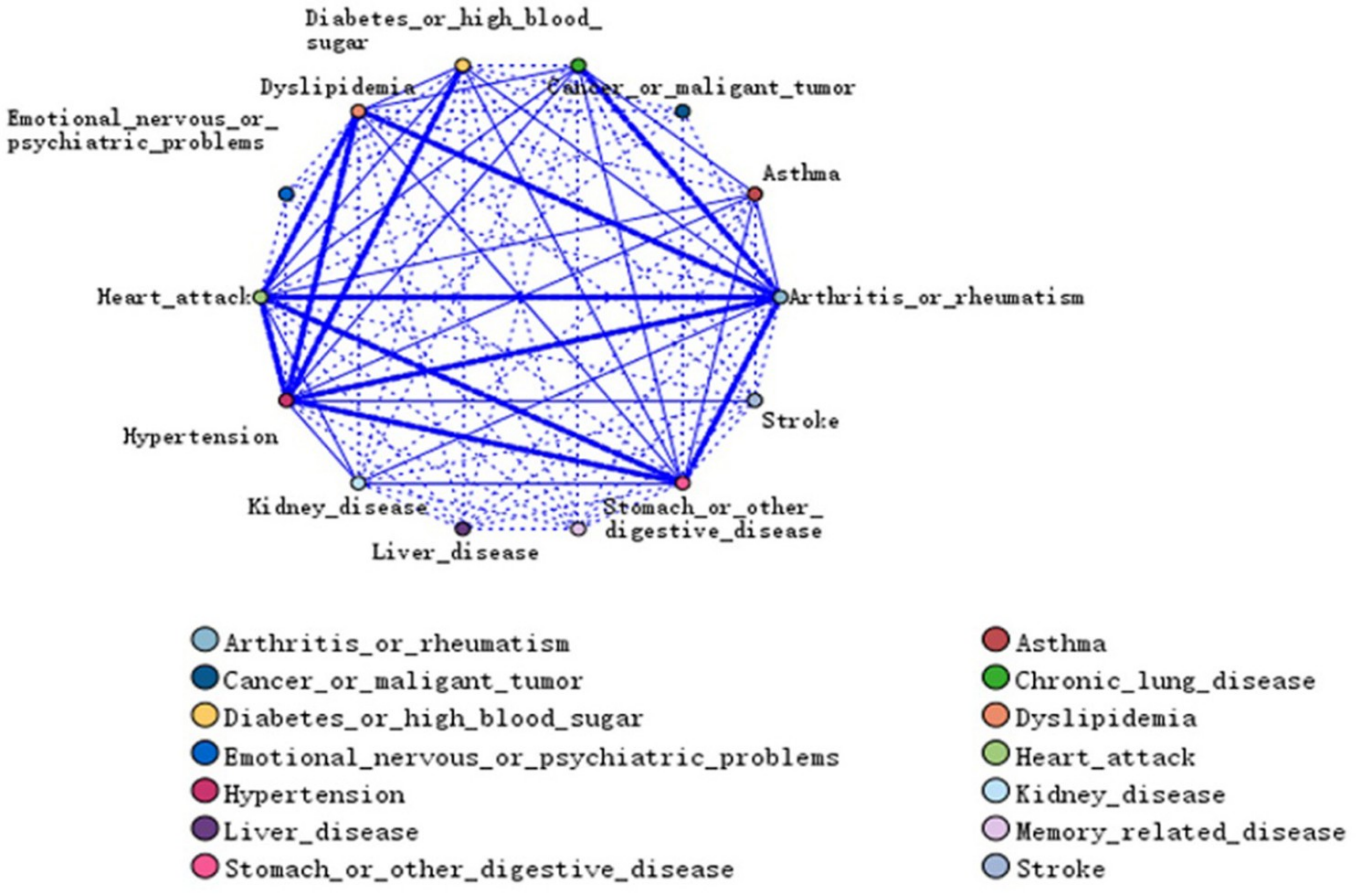
A web graph of associations between chronic diseases.

The web graph analysis showed strong association between [Stomach or other digestive disease/Arthritis or rheumatism], [Hypertension/Arthritis or rheumatism] and [Hypertension/Heart attack] (Fig 1). A total of 11 strong links are listed in S1 Table.

### Association among chronic diseases

Apriori algorithm was used to analyse the association among the chronic diseases. Under the conditions of min-support = 3%, min-confidence = 50%, and lift >1, and 11 association rules were selected (S2 Table). The support and confidence can also be used to show the prevalence of different multimorbidity patterns. For example, the prevalence rate of [Asthma/Chronic lung diseases] was 6.22%, among asthma patients and 63.77% in chronic lung disease patients.

With two left-hand-side, min-support, and min-confidence unchanged, 39 association rules were obtained, among which, 11 association rules with the one left-hand-side were excluded and nine association rules with confidence ≥ 60% and lift≥ 1.6 were selected (S2 Table).

With three left-hand-side, min-support, and min-confidence unchanged, 42 association rules were obtained, among which, 39 association rules with the two left-hand-side were excluded and four association rules were selected (S2 Table).

### Age-adjusted Charlson comorbidity index

Based on the AAC scores, the severity of multimorbidity was categorized into three grades: mild, with AAC score of 0–1; moderate, with AAC scores of 2–4; severe, with AAC scores of ≥5. The AAC in 5186 (53.41%) elderly individuals of the sample was mild, in 3775 (38.88%) was moderate, and in 749 (7.71%) was severe. The survival rates of the three groups were evaluated by rank-sum test and revealed that higher comorbidity score was significantly associated with lower 10-year survival rate (p < 0.001).

### Results of spatial autocorrelation

Moran’s I value is a weighted correlation coefficient similar to Pearson’s coefficient, which is obtained through the covariance of spatial units and requires the data to be normal. In this study, the prevalences of multimorbidity and chronic diseases were tested for normality and showed a normal distribution (p > 0.05).

On spatial analysis of multimorbidity, the following three most relevant multimorbidity patterns of association rules were selected: [Asthma/Chronic lung diseases], [Arthritis or rheumatism, Asthma/Chronic lung diseases] and [Dyslipidemia, Hypertension, Arthritis or rheumatism/Heart attack]. Results show that the prevalence of multimorbidity patterns for [Dyslipidemia, Hypertension, Arthritis or rheumatism/Heart attack] followed a distinctly positive correlation distribution in China, while the prevalence of the other two multimorbidity patterns showed random distributions without spatial correlation distribution in China (S3 Table).

**Fig 2:**
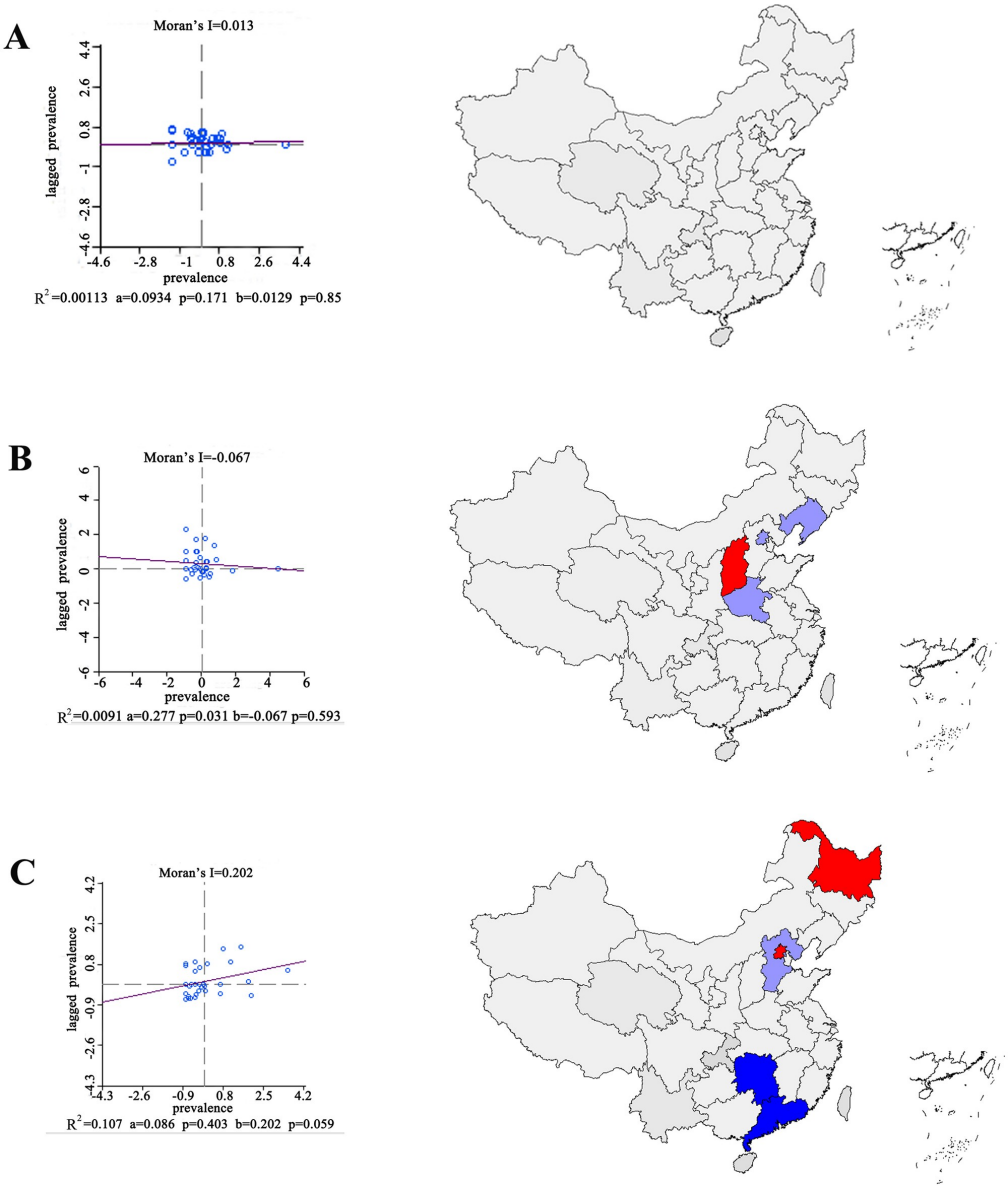
The result of local spatial autocorrelation of multimorbidity pattern. (The source of map: http://bzdt.ch.mnr.gov.cn/browse.html?picId=%224o28b0625501ad13015501ad2bfc0240%22). A, the result of local spatial autocorrelation of [Asthma / Chronic lung diseases]; B, the result of local spatial autocorrelation of [Arthritis of rheumatism, Asthma / Chronic lung diseases]; C, the result of local spatial autocorrelation of [Dyslipidemia, Hypertension, Arthritis or rheumatism / Heart attack].

Subsequently, we further analysed the spatial correlation of the prevalence of the three multimorbidity patterns in different regions, and the results are shown in Fig 2. The red areas are "high-high", the light blue areas are "low-high" and the dark blue areas are “low-low”.

## Discussion

In this study of multimorbidity in the elderly population in China, prevalence of most chronic diseases was higher in women, which is consistent with the recent report “*An Analysis Report of National Health Services Survey in China*” released in 2016. The possible reasons for this observation include higher life expectancy in females that results in longer period of illness, and also that among the elderly, the proportion of females who are widowed and live alone is higher than that of males, which may be associated with prominent psychological problems [20]. In other words, females have a survival advantage but no health advantage. Males have a higher prevalence of chronic lung diseases, stroke, kidney disease, and asthma that may be due to smoking, drinking, and other unhealthy lifestyles.

The overall prevalence of most chronic diseases is higher in the urban areas, while the prevalence of specific conditions such as stomach or other digestive diseases, arthritis or rheumatism, asthma, and emotional/nervous disorders or psychiatric problems is higher in the rural areas. This may be due to the fact that the general economic level of the elderly population in the urban areas is better with less work and more refined diet, while the elderly people in the rural areas have to work more and live in poor environmental conditions.

The prevalence of multimorbidity among the elderly in China is 49.46%. Fortin et al. [21] suggested that the estimation of the prevalence of multimorbidity was reliable only when the number of the types of chronic diseases included in the study is 12 or more. International Classification of Diseases (ICD) drives diseases into 22 catagories, including infections diseases, neoplasia, gestation and diseases of various systems. In this study, 14 types of chronic diseases were included, covering tumor, endocrine, mental health, neurology, circulatory, respiratory, gastrointestinal, musculoskeletal and urology/renal. In the meta-analysis of multimorbidities, the chronic diseases included in the 20 literatures were classified and counted, among which the 11 diseases with the most inclusion were included in our study. Therefore, the conclusions of our study are considered reliable [22]. Jiayi Gu et al. [23] in a survey of the prevalence of multimorbidity among the elderly in Chinese urban areas, found that the prevalence rate was 49.4%, which was similar to the results of this study. They also reported a higher prevalence of multimorbidity, in females (56.5%) than in males (45.7%), and the possible reasons were similar to those for the gender differences in prevalence of chronic diseases. They also reported that the prevalence of multimorbidity in urban areas is higher than that in rural areas, which is contrary to the results of another study in Jilin province, which may be related to the degree of economic development and aging patterns.

Results of the web graph can show the possible association between chronic diseases. In this study, the strongest link was [stomach or other digestive diseases/arthritis or rheumatism]. This may be because patients with arthritis take non-steroidal anti-inflammatory drugs, which can lead to gastrorrhagia and other diseases [24]. The second strongest link was [Hypertension/Arthritis or rheumatism]. In 2014, a survey conducted by Pauline Bocekxsstaens [25] in Belgium on people over 65 years old found that [hypertension/arthritis] was the most common patterns of chronic diseases. A longitudinal study lasting approximately 8 years found that patients with knee osteoarthritis were more likely to have hypertension [26]. This may be related to: 1) the pathological changes in the extracellular matrix of joints in patients with arthritis that results in decreased vascular elasticity and makes them more prone to hypertension; 2) although arthritis is not an inflammatory disease, it has chronic and mild inflammatory reactions, which play a role in the pathogenesis of cardiovascular diseases and hypertension. A research according to the German Claims Data reported that triads of the six most prevalent individual chronic conditions (hypertension, lipid metabolism disorders, chronic low back pain, diabetes mellitus, osteoarthritis and chronic ischemic heart disease) span the disease spectrum of 42% of the multimorbid sample [27].

The top three most relevant patterns from the association rule: [Asthma/Chronic lung diseases], [Asthma, Arthritis, or rheumatism/Chronic lung diseases], [Dyslipidemia, Hypertension, Arthritis or rheumatism/Heart attack] that were identified in this study have also been reported by other researchers. A study those participants from Beijing Medical Claim Data for Employees showed that the most prevalent disease pair was that of osteoarthritis and rheumatoid arthritis with hypertension. Ischaemic heart disease, hypertension, and osteoarthritis and rheumatoid arthritis constituted the most common triad combination [28]. A Netherlands study of 120, 000 general practice people reported that the most common combination of chronic diseases was chronic lung diseases and heart dysrhythmia [29].

The [Asthma/Chronic lung diseases] pattern has been noticed by many scholars in recent year [30, 31]. In a multi-centre study [32], the prevalence rate of Asthma-COPD overlap (ACO) in different age groups (20–44 years, 45–64 years, and 65–84 years) were 1.6%, 2.1%, and 4.5%, respectively, which was similar to the prevalence rate of [Asthma/Chronic lung diseases] multimorbidity pattern (6.17%) in this study.

[Asthma, Arthritis, or rheumatism/Chronic lung diseases] pattern is of great concern to researchers. Vered Bieber et al. [33] showed that patients with rheumatoid arthritis (RA) are more likely to experience COPD, because for both RA and COPD, smoking is a common risk factor [34, 35], and patients with both RA and COPD will have a heavier burden, and the mortality rate in these patients is significantly increased [36].

The risk of coronary artery disease complications in patients with RA is 1.5–2.0 times higher than that of the general population [37]. This study too reveals that 51.56% of the patients with Dyslipidemia, hypertension, and arthritis also experienced heart attack. This may be due to the existence of some common risk factors of Dyslipidemia, hypertension, arthritis, and heart disease, such as high fat diet and smoking.

Results of the AAC analysis showed a lower 10-year survival rate in the elderly with more chronic diseases, further highlighting the harmful impact of multimorbidity.

Finally, we conducted spatial autocorrelation analysis on the three selected multimorbidity patterns, which was helpful in observing the characteristics of multimorbidity in spatial distribution. The prevalence of the multimorbidity pattern [Asthma/Chronic lung diseases] was randomly distributed both globally and locally, indicating that the pattern was less affected by the cultural environment and more affected by the interaction between the two diseases. The prevalence of multimorbidity pattern [Asthma, Arthritis, or rheumatism/Chronic lung diseases] had “high-high” type in Shanxi and its surroundings, which may be related to the air quality in Shanxi province, where the dust-haze is severe. The prevalence of multimorbidity pattern [Hypertension, Dyslipidemia, Arthritis, or rheumatism/Heart attack] had “high-high” type in Heilongjiang province, a municipality of Beijing and its surroundings, and “low-low” type in Hunan province, in Guangdong province and its surroundings, which was roughly the same as the clustering area when the prevalences of hypertension, heart attack, and dyslipidemia were analysed separately. These results suggest that the environment, economy, and lifestyle factors have significant influence on this pattern.

In addition, in this study, the spatial analysis of chronic diseases and multimorbidity did not combine the economy, population and climate, so the spatial distribution could only be observed, but the reason for such distribution was difficult to be found. In the following studies, we will further analyze and explain the spatial distribution of chronic diseases and multimorbidity based on specific factors.

## Conclusions

In conclusion, the prevalence of chronic diseases and multimorbidity is high among the elderly population in China, and is higher in elderly women. The negative effects of multimorbidity are serious. Health departments should determine the characteristics of different multimorbidity patterns and develop corresponding treatment and medication guidelines in accordance with this information. In prevention and treatment of chronic diseases and multimorbidity, regional factors should be taken into consideration, and corresponding measures should be formulated more comprehensively according to the specific cultural and environmental factors in the area.

## Supporting information

S1 File Introduction of CHARLS database. (DOCX)

S1 Table The strong links of web graph. (DOCX)

S2 Table The results of association rules for relationship between chronic diseases with 1 left-hand-side. (DOCX)

S3 Table The result of global spatial autocorrelation of multimorbidity patterns. (DOCX)
