# Supplementary material for: Multimorbidity in the elderly in China based on the China Health and Retirement Longitudinal Study
Source: PLoS One. 2021 Aug 5;16(8):e0255908. doi: 10.1371/journal.pone.0255908 (PMC8341534; doi:10.1371/journal.pone.0255908)
Supplement: S1 File — (DOCX) [file pone.0255908.s001.docx]

China Health and Retirement Longitudinal Survey (CHARLS) is a large-scale interdisciplinary survey project hosted by Peking University, aiming to collect a set of high-quality micro-data representing families and individuals of middle-aged and elderly people aged 45 and above in China. CHARLS national baseline survey was launched in 2011, and followed up every two years in 28 province (autonomous region, municipality), and by 2015, the sample had been covered by a total of 23,000 of 1,240,000 households. The project adopted multi-stage sampling, and probability proportionate to size sampling (PPS) was adopted in both county/district and village residence sampling stages. CHARLS questionnaire include demographic backgrounds, family, health status and functioning, health care and insurance, work, retirement and pension, income, expenditures and assets, housing characteristics, etc., and it’s response rate and data quality are among the top of similar projects in the world, and the data has been widely used and recognized in academic.
