# Supplementary material for: Multimorbidity in the elderly in China based on the China Health and Retirement Longitudinal Study
Source: PLoS One. 2021 Aug 5;16(8):e0255908. doi: 10.1371/journal.pone.0255908 (PMC8341534; doi:10.1371/journal.pone.0255908)
Supplement: S1 Table — (DOCX) [file pone.0255908.s002.docx]

**S1 Table. The strong links of Web graph**

| Links | Field 1 | Field 2 |
| --- | --- | --- |
| 1377 | Stomach or other digestive disease | Arthritis or rheumatism |
| 1363 | Hypertension | Arthritis or rheumatism |
| 986 | Hypertension | Heart attack |
| 894 | Heart attack | Arthritis or rheumatism |
| 887 | Hypertension | Dyslipidemia |
| 794 | Hypertension | Stomach or other digestive disease |
| 699 | Heart attack | Stomach or other digestive disease |
| 654 | Dyslipidemia | Arthritis or rheumatism |
| 622 | Chronic lung diseases | Arthritis or rheumatism |
| 613 | Dyslipidemia | Heart attack |
| 558 | Hypertension | Diabetes or high blood sugar |
