# Supplementary material for: Multimorbidity in the elderly in China based on the China Health and Retirement Longitudinal Study
Source: PLoS One. 2021 Aug 5;16(8):e0255908. doi: 10.1371/journal.pone.0255908 (PMC8341534; doi:10.1371/journal.pone.0255908)
Supplement: S2 Table — (DOCX) [file pone.0255908.s003.docx]

**S2 Table. The results of association rules for relationship between chronic diseases with 1 left-hand-side**

|  | Left-hand-side | Right-hand-side | Support % | Confidence % | Lift |
| --- | --- | --- | --- | --- | --- |
| Left-hand-side=1 | Asthma | Chronic lung diseases | 6.17 | 63.77 | 5.15 |
|  | Stroke | Hypertension | 3.87 | 64.36 | 1.93 |
|  | Dyslipidemia | Hypertension | 15.37 | 59.45 | 1.79 |
|  | Diabetes or high blood sugar | Hypertension | 10.37 | 55.41 | 1.66 |
|  | Heart attack | Hypertension | 19.19 | 52.93 | 1.59 |
|  | Memory related disease | Hypertension | 3.44 | 51.80 | 1.56 |
|  | Stomach or other digestive diseases | Arthritis or rheumatism | 24.69 | 57.45 | 1.49 |
|  | Kidney disease | Arthritis or rheumatism | 7.77 | 56.10 | 1.45 |
|  | Chronic lung diseases | Arthritis or rheumatism | 12.39 | 51.70 | 1.34 |
|  | Asthma | Arthritis or rheumatism | 6.17 | 50.58 | 1.31 |
|  | Liver disease | Arthritis or rheumatism | 3.37 | 50.46 | 1.31 |
| Left-hand-side=2 | Asthma, ,arthritis or rheumatism | Chronic lung diseases | 3.12 | 64.03 | 5.17 |
|  | Diabetes or high blood sugar, dyslipidemia | Hypertension | 4.44 | 71.46 | 2.15 |
|  | Dyslipidemia, heart attack | Hypertension | 6.31 | 66.56 | 2.00 |
|  | Diabetes or high blood sugar, heart attack | Hypertension | 3.71 | 66.11 | 1.99 |
|  | Kidney disease, stomach or other digestive diseases | Arthritis or rheumatism | 3.48 | 69.53 | 1.80 |
|  | Chronic lung diseases, stomach or other digestive diseases | Arthritis or rheumatism | 4.52 | 68.57 | 1.77 |
|  | Heart attack, stomach or other digestive diseases | Arthritis or rheumatism | 7.20 | 63.95 | 1.66 |
|  | Hypertension, stomach or other digestive diseases | Arthritis or rheumatism | 8.18 | 62.09 | 1.61 |
|  | Dyslipidemia, stomach or other digestive diseases | Arthritis or rheumatism | 4.61 | 62.05 | 1.61 |
| Left-hand-side=3 | Dyslipidemia, hypertension, arthritis or rheumatism | Heart attack | 3.96 | 51.56 | 2.69 |
|  | Dyslipidemia, heart attack, arthritis or rheumatism | Stomach or other digestive diseases | 3.12 | 51.49 | 2.09 |
|  | Dyslipidemia, heart attack, arthritis or rheumatism | Hypertension | 3.12 | 65.35 | 1.96 |
|  | Heart attack, Stomach or other digestive diseases, Hypertension | Arthritis or rheumatism | 3.42 | 65.06 | 1.68 |
