# Supplementary material for: Multimorbidity in the elderly in China based on the China Health and Retirement Longitudinal Study
Source: PLoS One. 2021 Aug 5;16(8):e0255908. doi: 10.1371/journal.pone.0255908 (PMC8341534; doi:10.1371/journal.pone.0255908)
Supplement: S3 Table — (DOCX) [file pone.0255908.s004.docx]

**S3 Table. The result of global spatial autocorrelation of multimorbidity patterns**

| Multimorbidity patterns | Global Moran's I | *Z* | *P* |
| --- | --- | --- | --- |
| Asthma/Chronic lung diseases | 0.013 | 0.379 | 0.361 |
| Arthritis or rheumatism, Asthma/ Chronic lung diseases | -0.067 | -0.499 | 0.290 |
| Dyslipidaemia, Hypertension, Arthritis or rheumatism / Heart attack | 0.202 | 2.127 | 0.032 |
